# Influence of Ce, Nd, Eu and Tm Dopants on the Properties of InSe Monolayer: A First-Principles Study

**DOI:** 10.3390/nano11102707

**Published:** 2021-10-14

**Authors:** Zhi Xie, Limin Chen

**Affiliations:** College of Mechanical and Electronic Engineering, Fujian Agriculture and Forestry University, Fuzhou 350002, China; chenlm1998@163.com

**Keywords:** density functional theory, InSe, lanthanide, electronic structure

## Abstract

Doping of foreign atoms may substantially alter the properties of the host materials, in particular low-dimension materials, leading to many potential functional applications. Here, we perform density functional theory calculations of two-dimensional InSe materials with substitutional doping of lanthanide atoms (Ce, Nd, Eu, Tm) and investigate systematically their structural, magnetic, electronic and optical properties. The calculated formation energy shows that the substitutional doping of these lanthanide atoms is feasible in the InSe monolayer, and such doping is more favorable under Se-rich than In-rich conditions. As for the structure, doping of lanthanide atoms induces visible outward movement of the lanthanide atom and its surrounding Se atoms. The calculated total magnetic moments are 0.973, 2.948, 7.528 and 1.945 μB for the Ce-, Nd-, Eu-, and Tm-doped systems, respectively, which are mainly derived from lanthanide atoms. Further band structure calculations reveal that the Ce-doped InSe monolayer has n-type conductivity, while the Nd-doped InSe monolayer has p-type conductivity. The Eu- and Tm-doped systems are found to be diluted magnetic semiconductors. The calculated optical response of absorption in the four doping cases shows redshift to lower energy within the infrared range compared with the host InSe monolayer. These findings suggest that doping of lanthanide atoms may open up a new way of manipulating functionalities of InSe materials for low-dimension optoelectronics and spintronics applications.

## 1. Introduction

In recent years, owing to the distinguished mechanical, electronic, optical and magnetic properties, two-dimensional (2D) materials have attracted lots of research interests [1,2,3,4,5,6]. After the findings of graphene, a lot of 2D layered materials were investigated by experimental and theoretical methods, such as BN [3,4], phosphorene [5], and 2D transition metal dichalcogenides (TMDCs) [6,7,8,9]. Due to their novel properties, these 2D materials are considered to hold promising applications for future low-dimension electronic, spintronics and optoelectronic devices [10,11,12,13].

InSe is a new type of promising 2D material. In recent years, monolayer and few- layers of InSe were successfully prepared from the bulk counterpart [14,15]. In the experiment, InSe bulk is a direct bandgap semiconductor with a gap value of 1.26 eV [16], whereas InSe few-layers and monolayer are indirect semiconductors possessing tunable experimental band gaps from 1.3 eV to 2.2 eV depending on the number of layers [14,17,18]. In addition, many attractive properties were found in 2D InSe nanosheets. It was reported that photodetectors based upon few-layers InSe show photo-detection of broadband wavelength, and have high photo-responsivity and good gate tunable behavior [19,20]. The exceptional photoluminescence was disclosed in atomically layered InSe [21]. Because of the suppressed scattering of carriers in the 2D InSe system, the fabricated field-effect transistor made by InSe layers shows good mobility of 10^3^ cm^2^·V^−1^·s^−1^ degree [22]. The piezoelectricity effect in 2D InSe was also predicted to be in the same order of magnitude as that found in previously reported 2D piezoelectric materials [23].

Until now, 2D materials doped with nonmetal atoms, main group metal, and transition metal have been extensively explored [24,25,26,27,28,29]. For instance, Li et al. studied the single-layer InSe doped with VA and VIIA elements [25], and the doping effects on p/n conductivity of the system were discussed. Fu et al. investigated the substitutional and interstitial doping of B atoms in single-layer InSe via first-principles calculations [26], and found that the B interstitial doping triggers magnetic behavior of the system, while the substitutional doping system is nonmagnetic. Moreover, Sun et al. discovered the magnetism of As-doped 2D InSe can be manipulated by controlling different configurations of As doping [27]. The transition metal-doped 2D InSe materials were also studied using the density functional theory (DFT) scheme, and the magnetic and electronic properties of the systems were discussed [28,29].

In view of the abundant electronic configurations of 4f orbitals, lanthanide (Ln) atoms have demonstrated good magnetic and optical properties. The presence of Ln dopant was shown to be a useful measure to regulate the optical and magnetic properties of various bulk materials [30,31,32]. However, for the 2D systems, research efforts of Ln atom doping are still limited. Only a few research works were conducted in the phosphorene and 2D TMDCs [33,34,35,36]. For example, Bai et al. doped 2D MoS_2_ with Er in the experiment and realized the up-conversion and down-conversion fluorescence emission in the near-infrared region in the system. They also analyzed in detail the optical property, band structure and energy transfer mechanism of the doped systems [34]. In 2017, Ouma et al. investigated the Ln (Ce, Eu, Gd, Lu, Tm) doped MoS_2_ monolayer using the DFT calculations, and discussed their structural, electronic, optical and magnetic properties [35]. Recently, Obodo et al. conducted the DFT calculation of Ln-doped HfS_2_ monolayer and found the induced magnetic moment and redshift of absorption spectrum after doping [36]. However, to the best of our knowledge, up to now, the Ln doped 2D InSe systems have not been explored yet, despite that the complex and tunable electronic structures of the doped InSe systems may hold substantial promise for many potential applications. Here, we perform first-principles calculations of the Ln atoms (Ce, Nd, Eu, Tm) doped InSe monolayers, and investigate in detail the structure, magnetism, electronic and optical properties of the doped 2D InSe systems.

## 2. Calculation Methodology

In this study, all calculations are carried out using the Vienna ab initio simulation package (VASP) (version 5.4, VASP Software GmbH, Vienna, Austria) based on density functional theory. The electron-ion interactions are described by the projector augmented wave (PAW) pseudopotentials, and the exchange-correlation interactions are represented using the generalized gradient approximation (GGA) with Perdew-Burke-Ernzerhof (PBE) functional. A plane-wave basis with kinetic energy cutoff as 500 eV is used to expand the electronic wave functions. To eliminate the interactions between two neighboring periodic slabs, a 15 Å vacuum region is set in the *c* direction. A Monkhorst-Pack mesh of 5 × 5 × 1 is adopted for sampling the Brillouin zone. The convergence criteria of 10^−6^ eV are chosen for the energy calculation, and geometry relaxation is performed until the force on one atom is less than 0.01 eV/Å.

Since the standard GGA underestimates the bandgap of the calculated system, some improved methods such as hybrid functional, meta-GGA, and GGA + U were proposed to correct the band gap underestimation in GGA calculations for bulk and layer materials [37,38,39]. For lanthanide atoms, the strong electron correlation of localized 4f orbitals may play an important role in their electronic structure and magnetic properties, and the Hubbard U correction was successfully applied in several Ln doped 2D systems to improve the limitation of standard GGA for strongly correlated systems [33,35]. Hence, the GGA + U scheme is chosen for our study. However, there are no reported experimental or theoretical results to verify a fixed U value for Ln doped InSe monolayer, so the U values from 0.0 eV to 8.0 eV are employed to investigate the effect of U value on the description of 4f electrons. The calculation results indicate the U value has little effect on geometry structure and the main magnetism, but 4f states become localized from U > 4 eV, which is in accord with the previous theoretical findings of 4f orbitals using the GGA + U method [33,40]. Therefore, U = 6.0 eV is adopted in our study, which is similar to the former researches on lanthanide atom doped 2D systems [33,35,36].

## 3. Results and Discussion

### 3.1. Structure and Magnetic Properties

**Figure 1:**
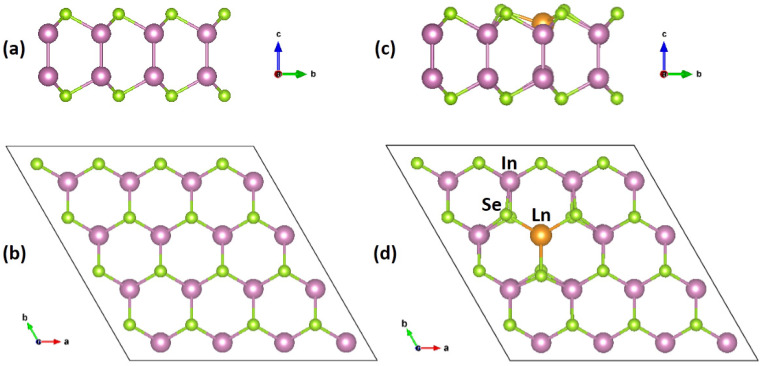
(**a**–**d**) Side view (**a**,**c**) and top view (**b**,**d**) of the pristine 4 × 4 × 1 InSe monolayer supercell (**a**,**b**) and the Ln-doped system (**c**,**d**). Ln stands for the lanthanide dopant atom (Ln = Ce, Nd, Eu, Tm). The In, Se, and Ln atoms are labeled in purple, green and yellow, respectively.

The side and top views of the pristine 4 × 4 × 1 InSe 2D supercell are displayed in Figure 1. The InSe monolayer has four stacking planes of Se–In–In–Se atoms with covalent bonding. A In atom occupies the core site of a tetrahedron, and it is surrounded by three nearest Se atoms and one adjacent In atom situated in the c direction. After geometry optimization, the obtained lattice parameters of the 4 × 4 × 1 InSe supercell are a = b = 16.30 Å, and the InSe monolayer has a thickness of 5.40 Å. The optimized bond lengths of In–In and In–Se are 2.82 Å and 2.69 Å, respectively. These geometry parameters of the optimized structure are consistent with the previously reported data [39,41]. Based upon the relaxed structure of the pristine InSe supercell, one In atom is substituted by an Ln atom to construct the Ln doped system, rendering the doping concentration of 3.125%.

Table 1 lists the optimized average Ln–Se bond length and Ln–In bond length in the Ln-doped systems, and they both are longer than the corresponding bonds in the pristine InSe system. This is due to the smaller radius of In atom than those of Ln atoms. The radius of In atom is 1.63 Å, while the radii of Ce, Nd, Eu, and Tm atoms are 1.82 Å, 1.81 Å, 2.08 Å, and 1.76 Å, respectively [42]. Figure 1c,d display a typical structure of Ln doped InSe monolayer after the geometry optimization. For the four substitutional doping cases, the larger Ln atomic radius and Ln–Se bond length induce outward movements of the Ln atom and its surrounding Se atoms.

To assess the feasibility of introducing Ln into the InSe monolayer, the formation energy (∆E_f_) of the doped system is obtained using the formula as follows [35,36,39]:

(1) $$\Delta E_{f}=E_{\mathrm{doped}}\;-\;E_{\mathrm{pristine}}\;-\;\mu_{\mathrm{Ln}}\;+\;\mu_{\mathrm{In}}$$

where E_pristine_ and E_doped_ are the calculated energies of the pristine and doped 4 × 4 × 1 InSe monolayer. μ_Ln_ represents the chemical potential of an Ln atom, which is determined by one lanthanide atom from its bulk phase. μ_In_ represents the chemical potential of an In atom, and it varies depending on the Se-rich or In-rich growth conditions. Under the In-rich (Se-poor) environment, μ_In_ is obtained as the energy of an In atom in the bulk phase. Under the Se-rich (In-poor) environment, μ_In_ is equal to the difference between the energy of a formula unit (InSe) from a 2D InSe monolayer and the chemical potential of an Se atom (μ_Se_), and μ_Se_ is represented by the energy of a Se atom in its bulk phase. In Table 1, it is noted that, for the four Ln-doped systems, their formation energies are all negative, indicating these Ln substitutional doping can be achieved under equilibrium conditions. In the four doping cases, both under the Se-rich and In-rich environments, the Ce-doped system is mostly favorable in energy, followed by the Nd, Tm, and Eu. Compared with the In-rich condition, the lower formation energies in the Se-rich condition demonstrate it is easier to introduce Ln dopants into the undoped InSe system under the Se-rich environment [28,29,39].

Table 1 also presents the total magnetic moments calculated for the four Ln-doped InSe systems, and they are 0.973, 2.948, 7.528 and 1.945 μB for the Ce, Nd, Eu and Tm doping cases, respectively. The magnetic moments are found to be introduced into InSe monolayer after the presence of Ln dopants, and they mainly originate from the Ln dopants since the obtained magnetic moments of Ce, Nd, Eu and Tm dopants are 0.998, 3.025, 7.027 and 1.889 μB, respectively. In the Ce- and Nd-doped systems, their smaller total magnetic moments than those of the Ln dopant atoms demonstrate that other atoms should contribute a few negative magnetic moments. In contrast, for the Eu- and Tm-doping cases, the relatively larger total magnetic moments than that of Ln dopant indicate there are some positive magnetic moments from other atoms.

**Figure 2:**
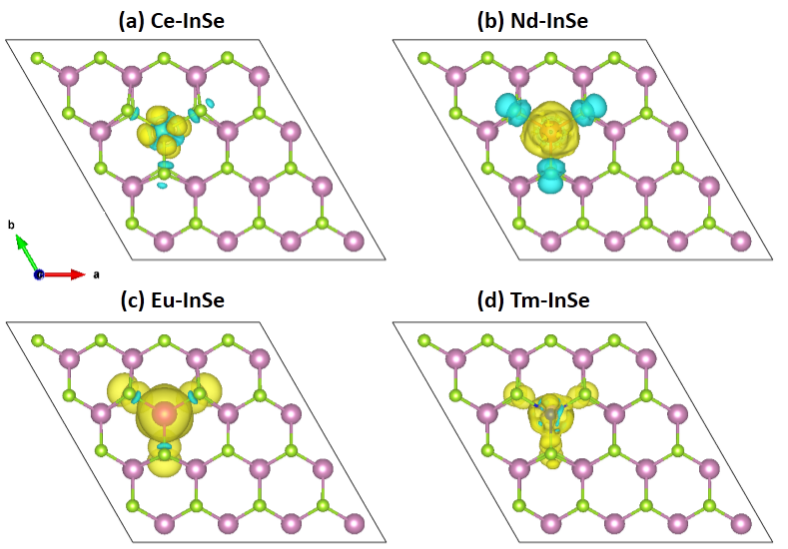
(**a**–**d**) Spin density of Ce-doped (**a**), Nd-doped (**b**), Eu-doped (**c**), and Tm-doped (**d**) InSe monolayer. The yellow and cyan isosurface represents the positive and negative spin density, respectively. The isosurface value is set to be 0.0003 e/Å^3^.

To further shed light on the magnetism in four Ln-doped 2D InSe systems, we calculated their spin densities, as shown in Figure 2, where the cyan and yellow isosurfaces represent the negative and positive spin-charge densities, respectively. It can be seen that for the Ce- and Nd-doped cases, their spin densities are mainly localized at the Ln dopant atoms, showing the characteristics of f electronic orbitals. In these two systems, the negative spin densities from the surrounding Se atoms of the Ln dopant atom are antiferromagnetically coupled to the Ln atom and show clearly the characteristics of p electronic orbitals. In the Eu- and Tm-doped cases, their main spin densities are also located at the dopant atoms, yet the spin densities of p orbital character from the neighboring Se atoms are positive, showing the ferromagnetic coupling to the dopant atom.

### 3.2. Band Structure and Electronic Property

**Figure 3:**
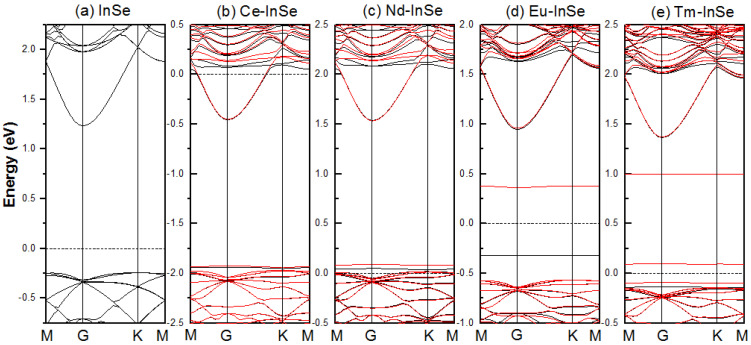
Band structures of the (**a**) pristine InSe monolayer and (**b**) Ce, (**c**) Nd, (**d**) Eu, and (**e**) Tm doped systems. The Fermi level is set to be zero. The black and red lines denote the spin−up and spin−down bands, respectively.

The band structures are calculated to probe the influence of the Ln dopant atom on the electronic structure of the InSe monolayer. For the undoped InSe monolayer, there is no difference between the spin-down and spin-up bands, and this should be ascribed to the non-magnetic nature of the InSe monolayer. As shown in Figure 3a, the undoped InSe monolayer shows the semiconducting character possessing an indirect bandgap of 1.48 eV, consistent with the reported theoretical values [39,41] and the experimental data [18]. Figure 3b–e plot band structures of Ce-, Nd-, Eu-, and Tm-doped systems, respectively. It can be seen that the spin-polarized character appears after doping the Ln atom, which is attributed to the presence of f electrons from the Ln dopants.

The Ce- and Nd-doped systems present metallic nature because the Fermi level crosses bands. The Ce-doped system exhibits n-type conductivity with the bottom of conduction bands intersected with the Fermi level, while the Nd-doped case is a p-type conductive system with the top of valence bands intersected with the Fermi level. In the Eu doping case, the band structures indicate that the system maintains a semiconducting character as the pristine InSe monolayer. Two impurity bands emerge in the bandgap of the undoped system after the Eu doping: one is a spin-up band lying at about −0.3 eV, and the other is a spin-down band lying at about 0.35 eV. The former constitutes a new valence band maximum (VBM) for the spin-up channel, while the latter forms a new conduction band minimum (CBM) for the spin-down channel. Hence, there appear two new band gaps for the Eu-doped InSe monolayer. The values of the bandgap are 0.93 eV and 1.26 eV for the spin-down and spin-up channels, respectively, and they both are less than the value (1.48 eV) of the pristine InSe monolayer. For the Tm-doped system, a similar semiconducting character occurs as the Eu doping case, and two new band gaps for the spin-down and spin-up channels are 0.18 eV and 1.49 eV.

**Figure 4:**
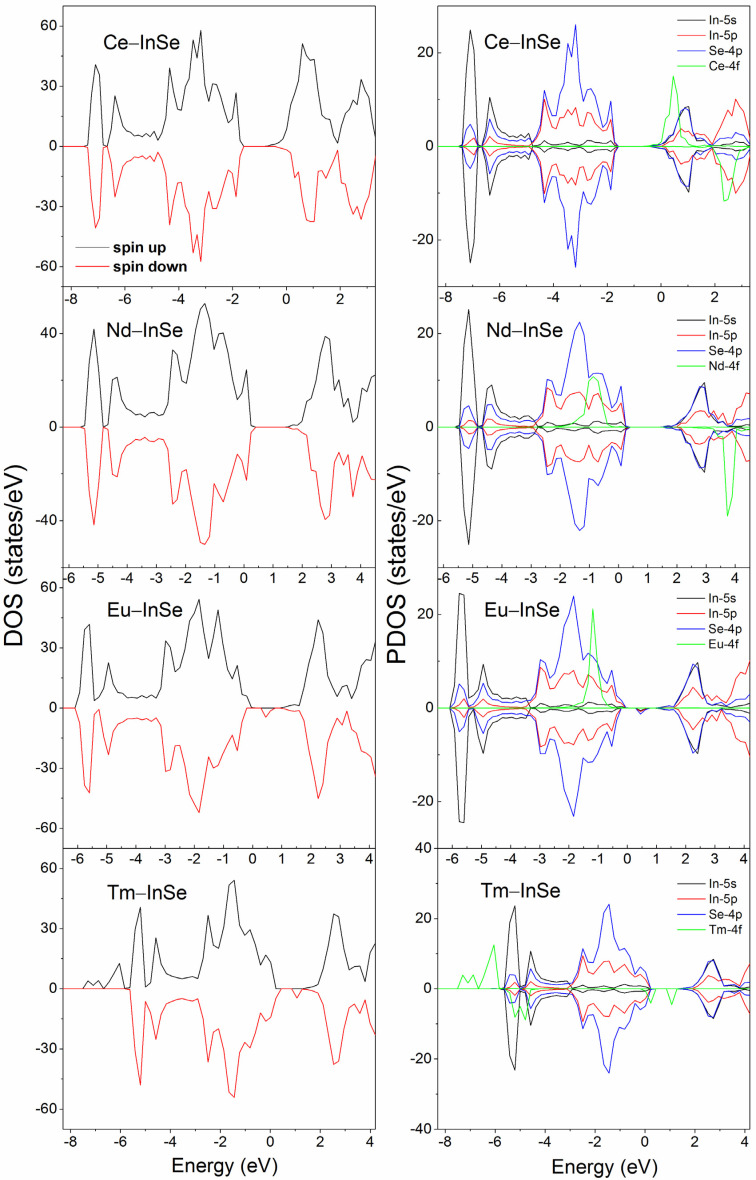
TDOS and PDOS of the four lanthanides (Ce, Nd, Eu, Tm) doped systems. The Fermi level is set to be zero.

To further analyze the critical impurity states and the states near the Fermi level, the total density of states (TDOS) and partial density of states (PDOS) of the four doped systems were calculated [43]. As shown in Figure 4, for the Ce-, Nd-, Eu-, and Tm-doped systems, their states corresponding to the valence bands and conduction bands retain the same components, which mainly consist of the s and p orbitals of In and Se atoms. In the Ce doping case, below the Fermi level, the imbalance of the spin-down and spin-up states is not obvious, and only a little asymmetric distribution of states emerges near the Fermi level, indicating the magnetism of this system is weak. This is consistent with the calculated results in Table 1, where the relatively small total magnetic moment (0.973 μB) for the Ce-doped system is shown, compared with those for Nd-, Eu-, and Tm-doped systems. From the PDOS, one can also note that the unoccupied states distributed from 0 eV to 2.5 eV are obviously asymmetric, which is ascribed to the presence of the main parts of Ce 4f states and their hybridization with the nearby p and s states. The critical states around the Fermi level stem from the In 5s, Se 4p and Ce 4f orbitals. For the Nd-doped system, the Nd 4f states appear in the regions of −1.5~−0.5 eV and 3.5~4 eV, leading to the asymmetry of spin-up and spin-down DOS located at these areas. These two spin channel states of Nd 4f orbitals lie in the separated regions above and below the Fermi level, so the magnetism of the Nd-doped system is relatively stronger compared with the Ce-doped case, which is also evidenced by the magnetic moment of 2.948 μB in Table 1. In the Nd doping case, the critical states around the Fermi level are Se 4p states and In 5p states. For the Eu-doped system, the Eu 4f states located in between −0.5 and −1.5 eV region induce the apparent asymmetric distribution of the DOS, and these states mainly contribute to the magnetic moment (7.528 μB) of the Eu-doped system. Moreover, the Se 4p and In 5p states distributed around −0.3 eV also show asymmetric features, and they correspond to the spin-up impurity band under the Fermi level (Figure 3). The states around 0.4 eV with spin-down character are related to the impurity band above the Fermi level, and these states are mostly contributed by Se 4p orbitals and some In 5p and In 5s orbitals. In the Tm doping case, the asymmetry of DOS is also caused by the f states. Below the Fermi level, there is the apparent asymmetric distribution of Tm 4f states which lie between −7.8 eV and −4.6 eV, and these states contribute to the main magnetic moment of the Tm-doped system. Above the Fermi level, there are some unoccupied Tm 4f states in the region of 0~2 eV, which correspond to the two impurity bands located in the bandgap of the undoped InSe monolayer.

### 3.3. Optical Properties

**Figure 5:**
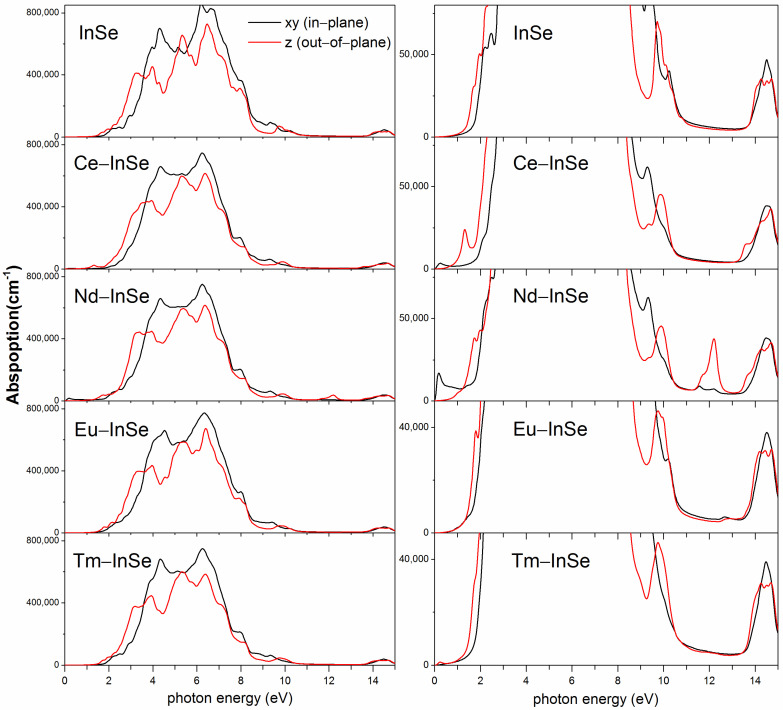
The calculated optical response of the absorption for the pristine InSe monolayer and the four lanthanides (Ce, Nd, Eu, Tm) doped systems, with E−field polarization, parallel to the xy−plane (black line) and out−of−plane z direction (red line).

The calculated optical properties for the undoped InSe monolayer and the Ln-doped systems are displayed in Figure 5. As for the linear regime, the dielectric function is given as,

ε (ω) = ε_1_ (ω) + iε_2_ (ω) (2)

where ε_1_ is the real part of the dielectric function, and ε_2_ is its imaginary part. The ε_2_ can be obtained from the calculation of transitions from the occupied states to unoccupied states, and the ε_1_ can be calculated using the Kramers–Kronig relation. The intensity of absorption can be determined from the dielectric function [44].

Figure 5 plots the absorption spectra of the undoped InSe monolayer and four Ln-doped systems, and the intensities of absorption for xy in-plane and out-of-plane z directions are provided. The pristine InSe shows no absorption for the energy region of 0~1.45 eV, and its absorption onset is at ~1.5 eV, which should be ascribed to electron transfer between the VBM and CBM states. This phenomenon is also in accordance with the results of band structures. In the Ce- and Nd-doping cases, the onset of strong absorption is near 1.5 eV and is about the same as that of the InSe monolayer. This feature can be understood from the analyses of the band structure and DOS of the doped systems. After the introduction of the Ce and Nd dopants, the states corresponding to the valence bands and conduction bands of the host InSe retain the same character and the energy gap between the two kinds of states are still about 1.5 eV. It is found that there are some weak absorptions in the low energy region of 0~1.5 eV for the Ce- and Nd-doped systems, which is due to the metallic properties of these two systems and similar to the results of Ln-doped MoS_2_ monolayer [35]. As for the Eu- and Tm-doped systems, the onset of strong absorption is also near 1.5 eV. In the Eu doping case, the weak absorption onset is red shifted to be ~1.0 eV, which is because the presence of impurity bands leads to the narrowed spin-down and spin-up band gaps of 0.93 eV and 1.26 eV respectively. In the Tm doping case, its weak absorption onset is red shifted to be ~0.2 eV, owing to the bandgap of 0.18 eV for the spin-down channel. It should be mentioned that the excitonic effects which are not included in the GGA + U calculations could have an influence on the optical properties, so further research using time-dependent DFT or Bethe-Salpeter equation methods can be worthy of attention [45,46].

## 4. Conclusions

The structural, magnetic, electronic and optical properties of Ln- (Ce, Nd, Eu, Tm) doped InSe systems were probed by DFT calculations with the GGA + U scheme. The calculated negative formation energies suggest the feasible substitutional doping of lanthanide atoms in the host InSe monolayer, and the doping prefers an Se-rich environment. All Ln-Se bonds are found to be elongated compared to the In-Se bonds, leading to outward movements of the lanthanide dopant atoms and their surrounding Se atoms. We also found that magnetic ground states emerge by introducing the lanthanide dopants, and the calculated total magnetic moments are 0.973, 2.948, 7.528 and 1.945 μB for Ce-, Nd-, Eu-, and Tm-doped systems, respectively. The spin densities are mainly located on the lanthanide dopants, and some negative spin densities from the neighboring Se atoms are antiferromagnetically coupled to the Ce and Nd atoms, while some positive spin densities are ferromagnetically coupled to the Eu and Tm atoms. Further calculation of electronic structure reveals that the Ce-doped system has n-type conductivity, while the Nd-doped system has p-type conductivity. The Eu-, and Tm-doped systems present diluted magnetic semiconducting features, and the former has new band gaps of 0.93 eV and 1.26 eV for the spin-down and spin-up parts, while the latter possesses bandgaps of 0.18 eV and 1.49 eV for the two spin channels, respectively. The Eu doping introduces impurity bands to the bandgap of the host InSe system, and they are derived from Se 4p orbitals and some In 5p and In 5s orbitals. While the impurity bands are from Tm 4f orbitals for the Tm doping case. The PDOS analysis shows that the 4f states from the lanthanide atoms induce asymmetric character in some energy regions, which lead to magnetism in the doped systems. The calculation of optical properties uncovers the redshift of absorption spectra in the four Ln-doped systems. These findings imply that doping of lanthanide atoms may open up an alternative avenue in tailoring properties of 2D InSe materials, holding potential applications for low-dimension optoelectronics and spintronics.

## Figures and Tables

**Table 1:** The average Ln–Se bond length (d_Ln–Se_) and Ln–In bond length (d_Ln–In_), formation energy (ΔE_f_), and total magnetic moment (M_tot_) and magnetic moment of the Ln dopant (M_Ln_) in the Ln-doped systems.

System	ΔE_f_ (eV)		M_tot_ (μB)	M_Ln_ (μB)	D_Ln–Se_ (Å)	D_Ln–In_ (Å)
	In-Rich	Se-Rich				
Ce-doped	−1.697	−2.743	0.973	0.998	2.83	3.29
Nd-doped	−1.379	−2.425	2.948	3.025	2.80	3.25
Eu-doped	−0.670	−1.716	7.528	7.027	2.87	3.45
Tm-doped	−0.878	−1.923	1.945	1.889	2.71	3.16
InSe	/	/	0	0	2.69	2.82

## Data Availability

All of the data reported in the paper are presented in the main text. Any other data will be provided on request.
